# Rhino-Orbital Cerebral Mucormycosis Causing Temporomandibular Joint Ankylosis: A Case Series of Two Patients

**DOI:** 10.7759/cureus.35194

**Published:** 2023-02-19

**Authors:** EJAZ A MOKHTAR, Qalbi Fatima, Shahrukh Akbar, Sharique Equbal, Ameera Salahudeen

**Affiliations:** 1 Oral and Maxillofacial Surgery, All India Institute of Medical Sciences, Patna, IND; 2 Periodontics, Buddha Institute of Dental Sciences and Hospital, Patna, IND; 3 Oral and Maxillofacial Surgery, College of Dental Sciences, Davangere, IND; 4 Oral and Maxillofacial Surgery, Buddha Institue of Dental Sciences, Patna, IND

**Keywords:** aggressive, mastoid air cells, temporal mucormycosis, septic arthritis, mucormycosis, tmj ankylosis

## Abstract

Temporomandibular joint ankylosis is caused by trauma, infection, autoimmune, inflammatory joint diseases, and several other minor causes. Mucormycosis causing temporomandibular joint ankylosis has not been reported. We report two cases of temporomandibular joint ankylosis caused by mucormycosis from 2018 to 2022. In both cases, the infection started in the maxilla, then progressed to orbit. After that, involvement of the mastoid process, styloid process, and base of the skull was observed in the first case, while in the second case, there was the involvement of the base of the skull and mandibular ramus. As the temporomandibular joint (TMJ) components are contiguous to the base of the skull, it got affected causing temporomandibular joint ankylosis. Mucormycosis was diagnosed by KOH mount. The smear examination showed aseptate hyphae at 90^0^. Histopathology examination further confirmed mucormycosis. Glycemic control was done by infusing Insulin (both Lantus and regular). The case was managed with aggressive debridement and interpositional arthroplasty with a buccal fat pad. Liposomal amphotericin infusion was also started pre-operatively and continued in the post-operative phase. After 4 years of follow-up, the patient is well and had adequate mouth opening.

Mucormycosis infection affecting the TMJ has been reported in the literature. However, this is the first report of a mucormycosis infection resulting in TMJ ankylosis in the literature. The infection should be aggressively managed. Reversals of an immunocompromised state, aggressive surgical management, and antifungal medication are the key factors for the success of the deadly fungal infection.

## Introduction

Mucormycosis is highly prevalent in India due to the presence of a humid climate and the high endemicity of diabetes. In the Indian population, the incidence is 80 times higher than in developed countries [1]. Rhinocerebral and pulmonary mucormycosis are the most common types of mucormycosis. Rhinocerebral mucormycosis affecting the skull base is an acute and deadly infection. The most common causes are an infection of the maxillary sinus and an ear infection. The base of osteomyelitis is usually caused by bacteria. Skull base osteomyelitis due to fungal origin is rare. The diagnosis of skull base osteomyelitis is usually late. The late involvement of the skull base is due to the deep extension of infection through the perivascular channel causing damage to the cancellous bone of the base of the skull [2]. Our patient was immunocompromised. We report a histopathologically proven case of mucormycosis of the maxilla, which subsequently involved the skull base and TMJ region causing TMJ ankylosis.

The involvement of the skull base occurs during the last stage of the disease. There are case reports in the English language literature discussing the involvement of TMJ in mucormycosis but mucormycosis causing TMJ ankylosis has not been reported. To our knowledge, this is the first case of mucormycosis of the maxilla with extensive involvement of the adjacent area and causing temporomandibular joint ankylosis.

## Case presentation

Case 1

**Figure 1:**
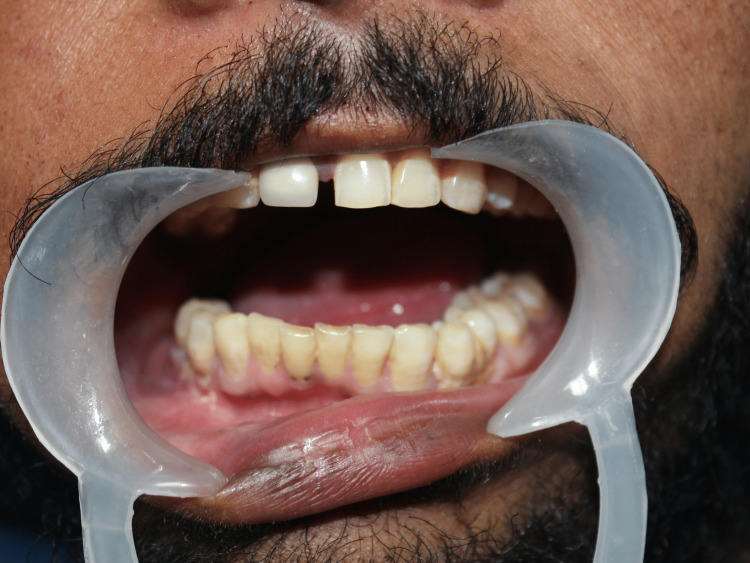
Decreased mouth opening pre-operatively

A 26-year-old patient presented with the chief complaint of inability to open the mouth widely for 2 years (Figure 1).

**Figure 2:**
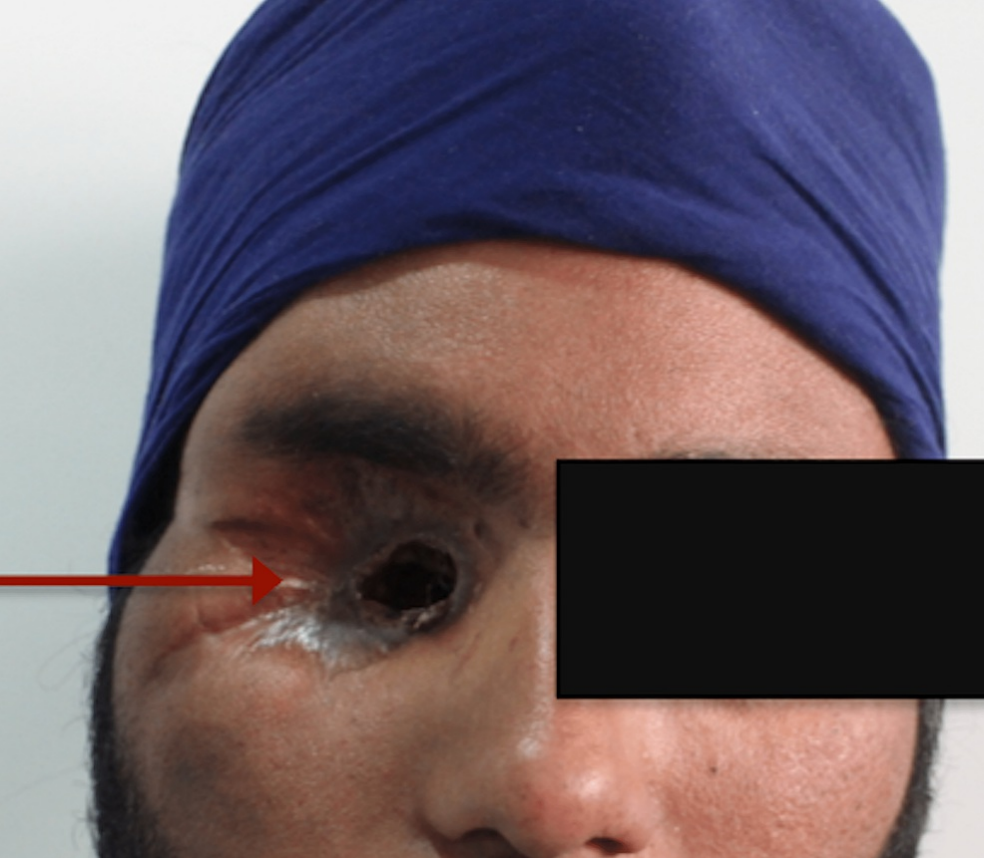
Arrow showing patient reported with exenterated orbit

He had a history of uncontrolled diabetes. His random blood sugar and Hb1Ac were 535 mg/dl and 14% respectively. An endocrinology consultation was taken and Insulin (both Lantus and regular) was started to control the elevated blood sugar levels. He had a previous history of trauma 5 years ago, after which he developed protrusion of the right eyeball. The history revealed a dilated pupil, restricted eye movement, and chemosis of the conjunctiva. Blackish necrotic tissue was seen on the right infraorbital region and orbital apex region. A smear sent from the periorbita revealed aseptate hypha. Orbital exenteration of the right side was performed (Figure 2).

**Figure 3:**
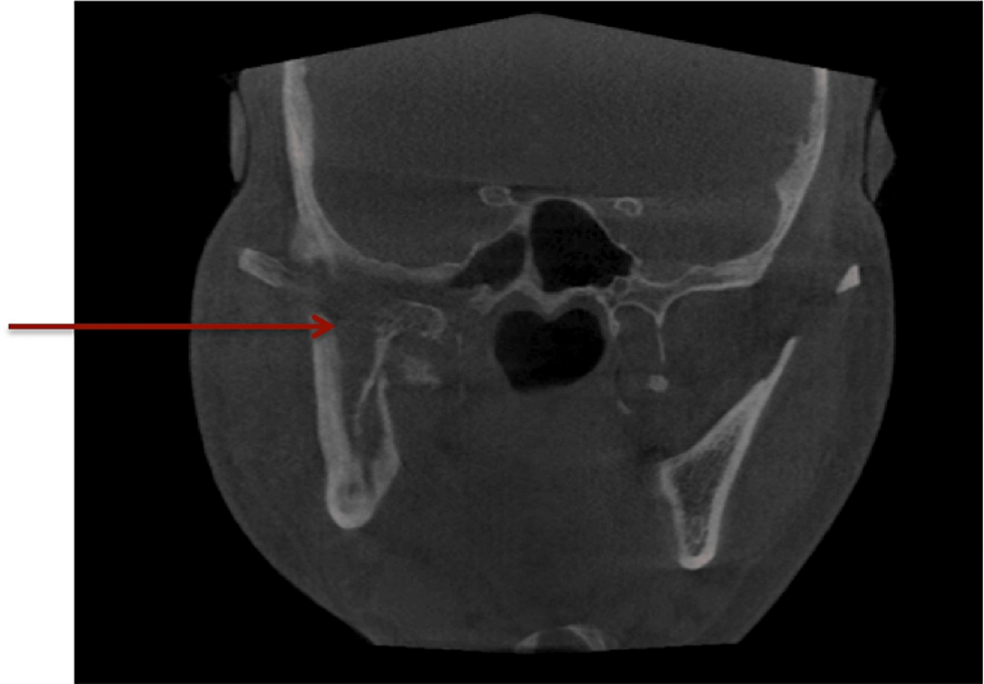
Arrow showing osteolytic changes in right side ramus, base of skull and mastoid

He had also undergone endoscopic debridement of the right paranasal sinus. Black necrotic tissue was seen. The histopathological report showed a case of invasive mucormycosis. A computed tomography scan showed osteomyelitis of the right-side base of the skull, condyle of the mandible, ramus region, and styloid process (Figure 3).

**Figure 4:**
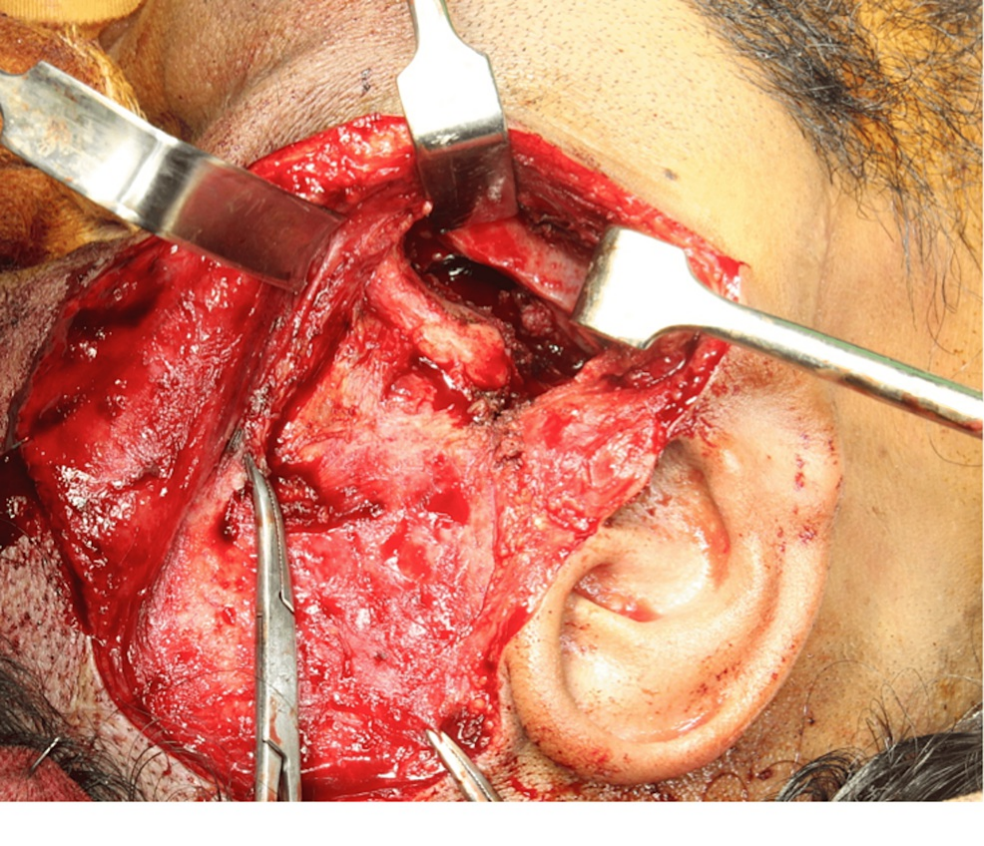
Standard Al kyat Bramley incision used to remove ankylotic mass

The diagnosis of TMJ ankylosis due to infective etiology was made. As there were chances of involvement of the brain, exploration of the area was planned and Neurosurgery was consulted The area was explored with a preauricular incision with temporal extension (Figure 4).

**Figure 5:**
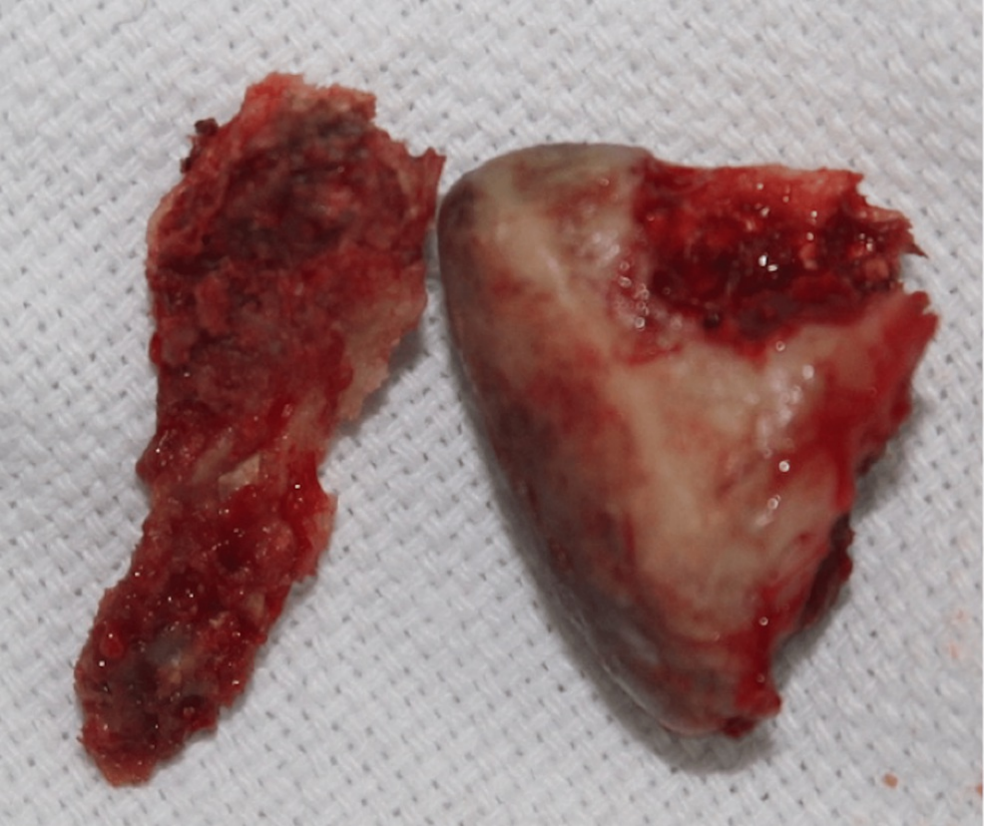
Removed condyle and styloid process

We found necrotic tissue in the temporomandibular joint region. Additionally, a fibrous band was seen between the osteolytic TMJ region and the glenoid fossa. Osteolytic changes were also seen in the styloid process and mastoid bone. The ankylotic region was released. Additionally, the involved styloid process, mastoid process, and all necrotic bone were removed (Figure 5).

Abdominal fat was used as interpositional material. Around 40 mm of mouth opening was achieved. The histopathology report showed aseptate hyphae consistent with mucormycosis. Liposomal amphotericin B was started. A total of 3 grams of intravenous amphotericin was given in 6 weeks. The daily dose was adjusted according to the renal status of the patient. The patient was kept on regular follow-up, and random blood sugar and Hb1Ac were monitored. Hb1Ac gradually improved from 15% to 6% in nine months. After four years of follow-up, the patient had adequate mouth opening with no recurrence.

Case 2

A 29-year-old male reported to the dental OPD with the chief complaint of inability to open his mouth widely. Eight months ago, he underwent endoscopic debridement of the right maxilla with right-side orbital exenteration for rhino cerebral mucormycosis.

**Figure 6:**
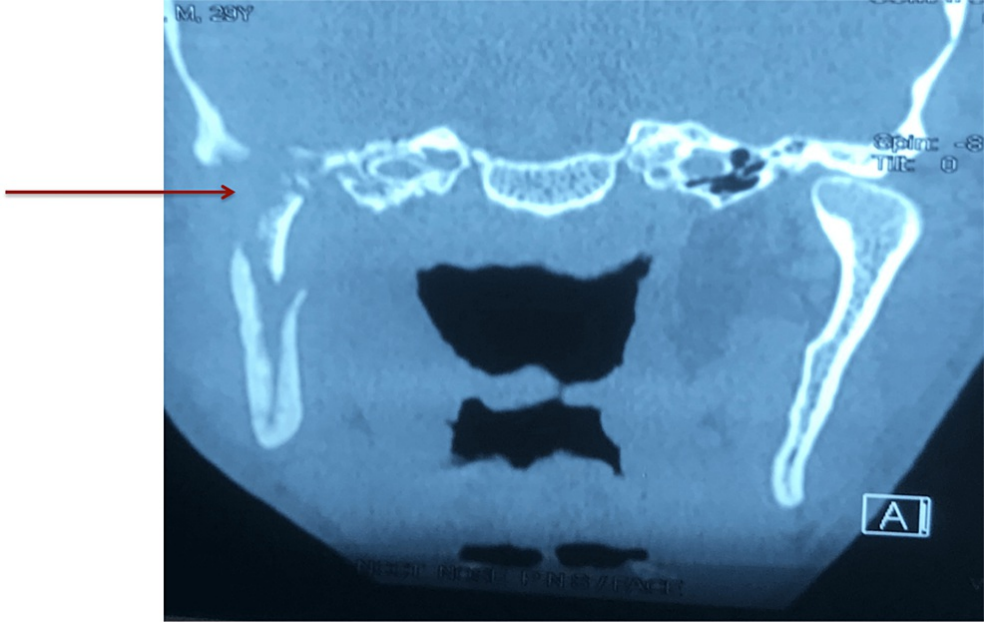
Arrow showing osteolytic changes in right side condyle and base of the skull

**Figure 7:**
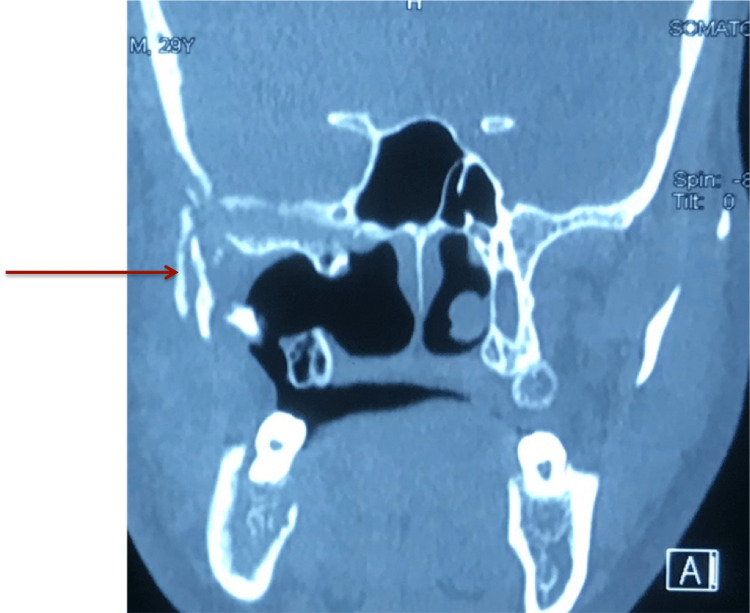
Arrow showing debrided right maxilla with osteolytic changes in the base of the skull

 He also had a previous history of COVID-19 six months before developing rhinocerebral mucormycosis. He had cone beam computed tomography (CBCT), which showed an osteolytic area involving the right side condylar region and squamous temporal bone and skull base (Figure 6, 7).

Magnetic resonance imaging (MRI) was done to rule out an intracranial extension. The patient was planned for interpositional arthroplasty with debridement of the skull base, but the patient was lost to follow-up.

## Discussion

The etiology of TMJ ankylosis can be broadly subdivided into major categories: post-traumatic and post-infective. Septic arthritis, rheumatoid arthritis, osteoarthritis, and various systemic infections are the main cause of TMJ ankylosis. Zhangcai [3] reported a case of mucormycosis of the temporal bone with extensive involvement of the infratemporal, parapharyngeal, and retropharyngeal spaces, and also involved the temporomandibular joint. However, the pathology has not resulted in TMJ ankylosis. Hall and Farrior classified temporal bone infection into non-invasive, acute invasive, and chronic invasive based on duration. In our first case, TMJ ankylosis occurred after four years of initial infection. Hence, it was a case of chronic invasive mucormycosis. There are only ten reported cases of mucormycosis involving temporal bone [4,5,6] in English language literature so far. However, only one case discussed TMJ involvement [2] but none has discussed rhinocerebral mucormycosis resulting in TMJ ankylosis.

The pathological involvement of the TMJ can be due to bacterial, viral, and fungal microorganisms [7]. The microorganism can enter the TMJ through 3 routes: 1) hematogenous, 2) contiguous, and 3) direct inoculation [8]. Cai et al reported 40 cases and showed that most organisms spread hematogenously [9]. Bacterial infections are most commonly seen causing TMJ ankylosis. The bacteria most commonly isolated in TMJ ankylosis is *Staphylococcus aureus. *The infection of the joint initiates an inflammatory response in the joint and subsequent joint destruction. The sequelae are the development of increased intraarticular pressure which subsequently leads to decreased flow, ischemia, and necrosis of cartilage leading to TMJ ankylosis [10]. In both of our cases, the maxilla was affected initially. The residual infection got reactivated when the patient's immune system became weak. Subsequently, the infection spread to adjacent sites affecting the mastoid bone, styloid process, and skull base. TMJ was affected by the direct extension of the infection from the skull base in both of the cases. The involvement of the right-side maxilla and right side of the TMJ infers that the infection has spread through direct extension and not through blood, which is the most common mode of spread of septic arthritis. 

Imaging plays an integral role in the diagnosis. In a computer tomography scan, this presents as an osteolytic lesion involving the TMJ area as seen in our case. Treatment consisted of reversal of the underlying condition, aggressive surgical management, and antifungal protocol [11,12]. We managed our case with both radical mastoidectomy, resection of the styloid process, removal of the ostelolytic and necrotic area, and removal of the ankylotic area. Post-operatively, the patient was put on an antifungal protocol (IV Amphotericin B). Around 3 grams of total amphotericin was administered. After 4 years of follow-up, the patient is well and had adequate mouth opening.

## Conclusions

In conclusion, this is the first report of mucormycosis causing temporomandibular joint ankylosis. The case highlights the inclusion of mucormycosis as a cause of TMJ ankylosis and the direct spread of infection from the contiguous site. It can be successfully managed with both medical management, and aggressive debridement and interpositional arthroplasty. During management, special attention should be given to the adjacent area of the temporomandibular joint, and the necrotic region should be completely removed.
